# A Case of Adult T-Cell Leukemia/Lymphoma Complicated with Bilateral Chylothorax

**DOI:** 10.1155/2019/8357893

**Published:** 2019-02-17

**Authors:** Satoko Kako, Satoru Joshita, Akemi Matsuo, Kenji Kawaguchi, Takeji Umemura, Eiji Tanaka

**Affiliations:** ^1^Department of Medicine, Division of Gastroenterology and Hepatology, Shinshu University School of Medicine, Matsumoto, Japan; ^2^Department of Respiratory Medicine, Shinonoi General Hospital, Nagano, Japan; ^3^Research Center for Next Generation Medicine, Shinshu University, Matsumoto, Japan; ^4^Department of Diagnostic Pathology, Shinonoi General Hospital, Nagano, Japan

## Abstract

We present the case of a 74-year-old Japanese woman who presented with dyspnea, a palpable right breast mass, and swollen right axillary lymph node. Imaging studies revealed bilateral pleural effusion and systemic lymph adenopathy and pleural fluid study showed high levels of triglycerides. A right inguinal lymph node biopsy disclosed malignant lymphoma cells that were human T-cell leukemia virus type 1 (HTLV-1) provirus DNA-positive, a condition endemic to patient's birthplace, by the Southern blot hybridization method. She was diagnosed as having adult T-cell leukemia/lymphoma (ATL) with chylothorax. After commencing chemotherapy for ATL, her chylothorax disappeared and swollen lymph nodes reduced remarkably, indicating an association between the chylothorax and ATL. Bilateral chylothorax is a relatively rare condition associated with such nontraumatic causes as ATL. Clinicians should therefore bear chylothorax in mind when encountering patients with pleural effusion. A detailed medical history can also enable prompt diagnosis and appropriate treatment.

## 1. Introduction

Chylothorax is caused by a disruption or obstruction of the thoracic duct or its tributaries that results in chyle leakage into the pleural space. Chylothorax fluid typically contains a high concentration of triglycerides in the form of chylomicrons if the patient has dietary fat consumption [1]. Although chylothorax has a wide range of etiologies [2–6], malignancy-associated chylothorax is not uncommon.

We herein describe the rare case of a female patient with adult T-cell leukemia/lymphoma (ATL) who showed bilateral chylothorax that became improved during ATL chemotherapy.

## 2. Case Presentation

A 74-year-old Japanese woman was referred to our hospital with dyspnea, a palpable mass in the right breast, and an enlarged lymph node in the right axilla that had worsened during the two months before admission. History taking revealed that she had moved from her birthplace in Kumamoto prefecture of southwestern Japan to Nagano prefecture after marriage. She had no other remarkable history of disease, transfusion, medication, or drug abuse.

On presentation, patient's body temperature was 37.2°C with a heart rate of 127 bpm and peripheral artery oxygen saturation of 92% in ambient air. Her vesicular sounds decreased without crackling on chest auscultation. Physical examination revealed a distended abdomen without hepatosplenomegaly. Systemic lymphadenopathy and pretibial edema pitting were noted.

**Figure 1:**
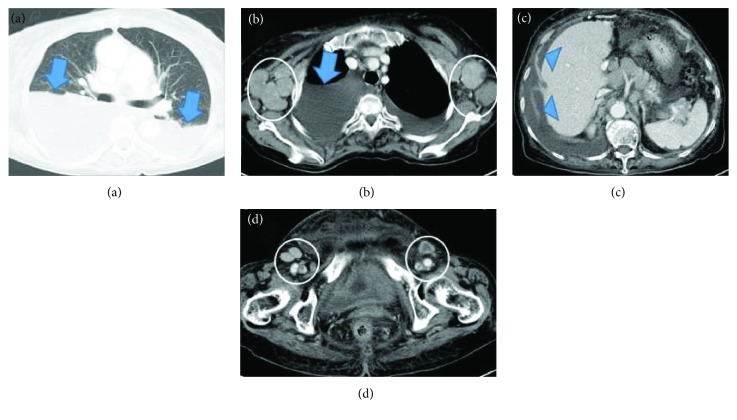
Computed tomography of the chest and abdomen depicted bilateral pleural effusion (arrows) (a, b) and ascites (arrowheads) (c). Axillary lymph adenopathy (circles) (b) and inguinal lymph adenopathy (circles) (d) were present.

**Figure 2:**
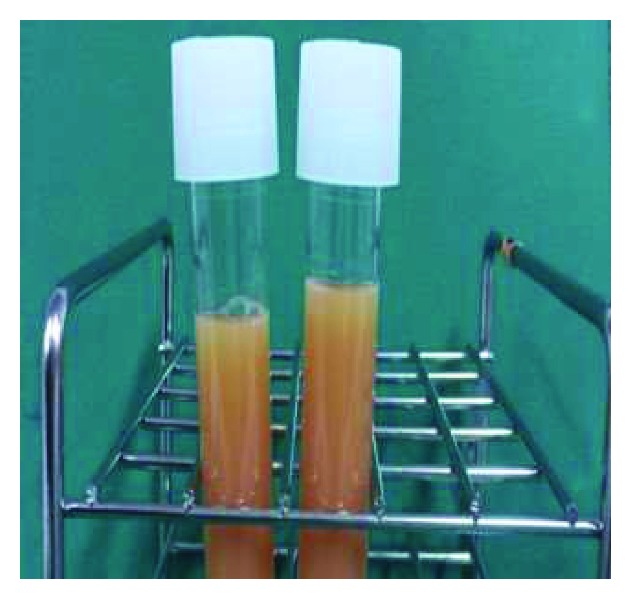
Appearance of bilateral pleural effusion of the chest was lightly bloody and chylous from each thoracic cavity.

**Figure 3:**
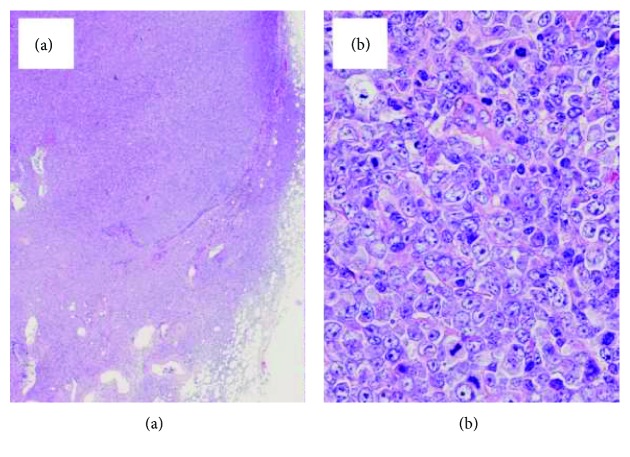
Histological findings of the right inguinal lymph node displayed diffuse architectural effacement (a). Neoplastic cells were medium- to large-sized with nuclear pleomorphism (b).

**Figure 4:**
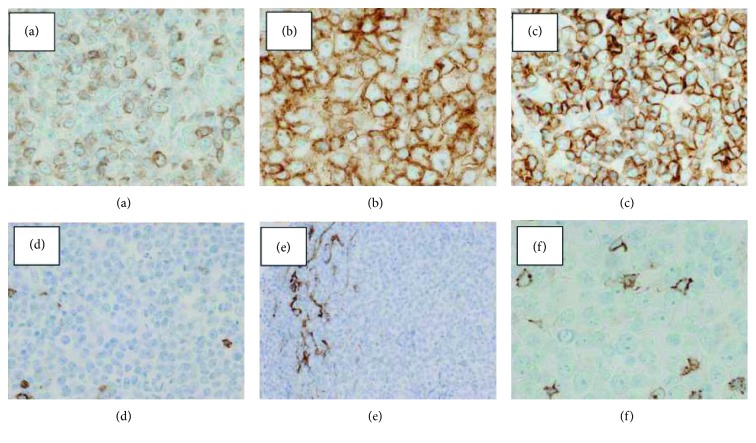
Tumor cells were CD3+ (a), CD4+ (b), CD5+ (c), CD8- (d), CD 20- (e), and CD21- (f) on immunohistochemistry.

**Figure 5:**
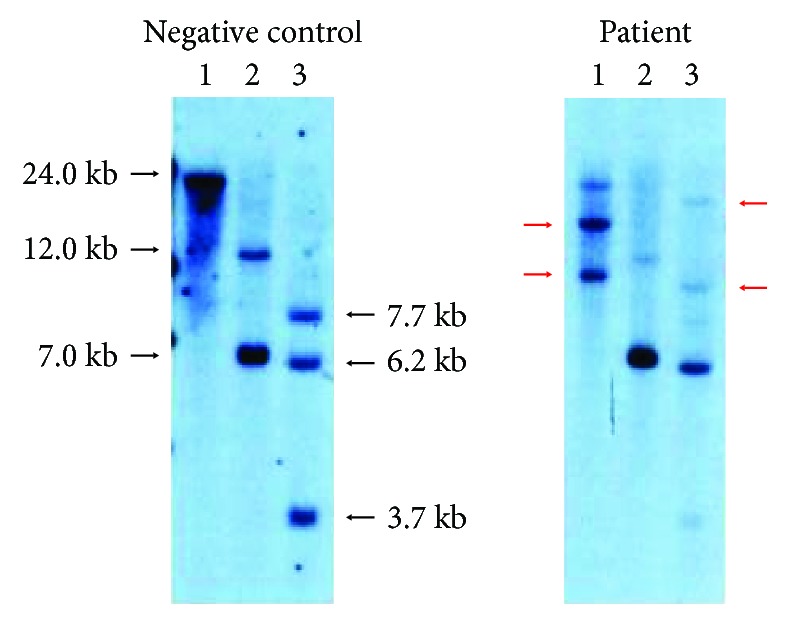
Southern blot analysis of HTLV-1 provirus DNA depicted two bands (red arrows) in after BamH-I digestion and two bands (red arrows) after Hind III digestion. 1: BamH-I; 2: EcoR-V; 3: Hind III.

Blood examination (Table 1) disclosed a lymphocyte count of 680/*μ*L and less than 1% morphological flower cells. Peripheral laboratory tests were as follows: aspartate aminotransferase, 37 U/L; alanine aminotransferase, 6 U/L; lactate dehydrogenase (LDH), 622 U/L; total bilirubin, 1.5 mg/dL; soluble IL-2 receptor, 27,500 U/mL (normal range: 135-421 U/mL); and calcium, 12.9 mg/dL. HTLV-1 antibody was positive. A contrast-enhanced computed tomography (CT) scan of the chest and the abdomen revealed bilateral pleural effusion and ascites with lymphadenopathy (Figure 1). Bilateral pleural effusion samples appeared chylous (Figure 2) with high triglyceride concentrations (Table 2) and class III cytology. A biopsy obtained from the right inguinal lymph node showed diffuse infiltration of moderate- to large-sized lymphoid cells with a pleomorphic nucleus and prominent nucleoli (Figure 3) that were CD3+, CD4+, CD5+, CD8-, CD20-, and CD21- on immunohistochemistry (Figure 4). Two monoclonal bands for HTLV-1 provirus DNA were observed in lymph node specimens by Southern blot hybridization analysis (Figure 5). Tumor cell infiltration into the bone marrow was negative in an aspiration biopsy. Based on these findings, the patient was diagnosed as having lymphomatous ATL.

**Figure 6:**
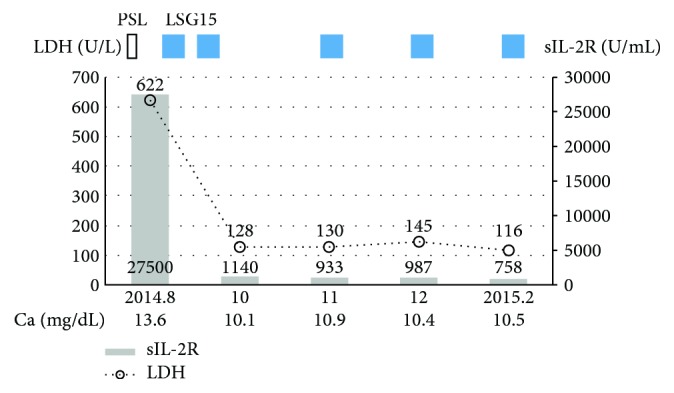
Clinical course of the patient. Abbreviations: PSL: prednisolone; LDH: lactate dehydrogenase; sIL-2R: soluble interleukin-2 receptor.

The clinical course of the patient is summarized in Figure 6. High-dose methylprednisolone therapy was deemed ineffective for her chylothorax since continuous pleural effusion drainage of 500 to 1,000 mL/day was necessary. The patient was soon shifted to a reduced-dose LSG15 chemotherapy regimen with prophylactic hydration, rasburicase, and bisphosphonate. After the first course of modified LSG15 (consisting of vincristine, cyclophosphamide, doxorubicin, prednisolone, vindesine, etoposide, ranimustine, and carboplatin) [7], she required pleural effusion drainage of less than 200 mL/day. Her systemic lymphadenopathy and pleural effusion disappeared over the two subsequent LSG15 treatment courses as confirmed by CT and normalization of LDH and calcium levels.

## 3. Discussion

An uncommon lymphoid neoplasm, ATL is considered a peripheral T-cell neoplasm associated with infection by the human T-lymphotropic virus type I (HTLV-I) according to the 2016 World Health Organization classification [8, 9]. Hence, the incidence of ATL depends on the prevalence of HTLV-I infection. HTLV-1 infection is specifically endemic in several islands in southwestern Japan [10], with most affected patients living or originating from these areas. As geographical displacement is frequent in Japan, a careful medical history that includes birthplace and upbringing may be helpful for the differential diagnosis of HTLV-1 infection, as demonstrated in this case.

To our knowledge, no case of ATL and bilateral chylothorax has been published to date. Chylothorax requires differential diagnosis from cholesterol effusions because both classically have a milky or opalescent appearance. It is especially important to distinguish the two since their etiology and treatment differ as well. Pleural fluid analysis can identify the characteristically elevated triglyceride levels of chylothorax as seen in our patient. Once diagnosed, chylothorax can be categorized as traumatic or nontraumatic [2]. Regarding the former, surgical procedures near the thoracic duct account for the majority of cases by disrupting the thoracic duct or tearing lymphatic tributaries [3, 11]. Accordingly, clinicians should directly inquire about any previous surgical interventions at this site. Concerning nontraumatic chylothorax, malignancies such as lymphoma, chronic lymphocytic leukemia, and metastatic cancer are the leading causes [2] and occasionally complicate diagnosis. Moreover, chylothorax is most common on the left side owing to the position of the thoracic duct, while bilateral chylothorax is rare but should always be considered. With regard to the mechanism of chylothorax in this case, we speculated an association with thoracic duct obstruction due to direct ATL cell invasion into the duct or possibly lymphadenopathy in the thorax because the chylothorax disappeared after the commencement of chemotherapy for ATL.

Following diagnosis by pleural fluid analysis, imaging techniques can help to identify the site and mechanisms of chyle leakage in most chylothorax cases. The gold standard for localizing lymph leakage is direct visualization at surgery or by lymphangiography. Specifically, case series have indicated that noncontrast magnetic resonance (MR) lymphangiography provides accurate visualization of the central thoracic lymphatics and sites of chyle leakage [12]. Dynamic contrast-enhanced MR lymphangiography with intranodal injection of a gadolinium-based contrast agent into the inguinal lymph nodes has also been reported to provide good spatial and temporal resolution for visualizing the anatomy and flow characteristics of the central lymphatic system [13]. However, neither of these approaches is 100% sensitive since the leakage may be too slow or diffuse to visualize. Additionally, these techniques are not available in many institutions and may be contraindicated in patients with systemic conditions. Thereafter, afflicted patients should begin prompt, appropriate chemotherapy for an improved prognosis.

In conclusion, bilateral chylothorax is a relatively rare condition that can be associated with such nontraumatic causes as ATL. Clinicians should consider chylothorax when encountering pleural effusion and commence treatment promptly after careful history taking.

## Figures and Tables

**Table 1:** Laboratory findings.

White blood cells	5,700/*μ*L	Total protein	6.7 g/dL
Neutrophils	77.9%	Albumin	3.3 g/dL
Eosinophils	0.2%	Total bilirubin	1.5 mg/dL
Basophils	0.4%	Na	140 mEq/L
Monocytes	9.6%	K	4.2 mEq/L
Lymphocytes	11.9%	Cl	102 mEq/L
Red blood cells	533 × 10^4^/*μ*L	Ca	13.6 mg/dL
Hemoglobin	17.3 g/dL	BUN	13 mg/dL
Hematocrit	50.8%	Creatinine	0.66 mg/dL
Platelet count	23.2 × 10^4^/*μ*L	Uric acid	9.3 mg/dL
Coagulation		Total cholesterol	227 mg/dL
PT	11.3 sec	Glucose	112 mg/dL
APTT	36.0 sec	Amylase	22 IU/L
Fibrinogen	201 mg/dL	CRP	0.52 mg/dL
FDP	19.9 *μ*g/mL	TPHA	Negative
AST	37 U/L	HBs Ag	Negative
ALT	6 U/L	HCV Ab	Negative
LDH	622 U/L	HIV Ab	Negative
ALP	222 U/L	HTLV-1 Ab	Positive
GGT	18 U/L	sIL-2R	27,500 U/mL

Abbreviations: PT: prothrombin time; APTT: activated partial thromboplastin time; FDP: fibrin/fibrinogen degradation product; AST: aspartate aminotransferase; ALT: alanine aminotransferase; LDH: lactate dehydrogenase; ALP: alkaline phosphatase; GGT: *γ*-glutamyltransferase; BUN: blood urea nitrogen; CRP: C-reactive protein; TPHA: treponema pallidum hemagglutination test; HBs Ag: hepatitis B virus s antigen; HCV Ab: hepatitis C virus antibody; HIV Ab: human immunodeficiency virus antibody; HTLV-1 Ab: human T-cell leukemia virus type 1 antibody; sIL-2R: soluble interleukin-2 receptor.

**Table 2:** Pleural effusion properties.

	Left	Right
Appearance	Light blood, chylous	Light blood, chylous
Cell count (/*μ*L)	904	349
Lymphocytes : granulocytes (%)	87 : 13	99 : 1
Protein (g/mL)	3.6	3.3
Glucose (mg/dL)	112	93
Specific gravity	1.032	1.030
pH	8.0	8.0
Lactate dehydrogenase (U/L)	261	278
Total cholesterol (mg/dL)	102	
Triglycerides (mg/dL)	369	337
Carcinoembryonic antigen (ng/mL)	1.1	
Adenosine deaminase (U/L)	25.2	
Cytology	Class III^∗^	Class III^∗^

^∗^ Cytology suggestive of, but not conclusive for, malignancy.
